# Disomic Inheritance and Segregation Distortion of SSR Markers in Two Populations of *Cynodon dactylon* (L.) Pers. var. *dactylon*

**DOI:** 10.1371/journal.pone.0136332

**Published:** 2015-08-21

**Authors:** Yuanwen Guo, Yanqi Wu, Jeff A. Anderson, Justin Q. Moss, Lan Zhu

**Affiliations:** 1 Department of Plant and Soil Sciences, Oklahoma State University, Stillwater, Oklahoma, United States of America; 2 Department of Horticulture and Landscape Architecture, Oklahoma State University, Stillwater, Oklahoma, United States of America; 3 Department of Statistics, Oklahoma State University, Stillwater, Oklahoma, United States of America; USDA, UNITED STATES

## Abstract

Common bermudagrass [*C*. *dactylon* (L.) Pers. var. *dactylon*] is economically and environmentally the most important member among *Cynodon* species because of its extensive use for turf, forage and soil erosion control in the world. However, information regarding the inheritance within the taxon is limited. Accordingly, the objective of this study was to determine qualitative inheritance mode in common bermudagrass. Two tetraploid (2n = 4x = 36), first-generation selfed (S1) populations, 228 progenies of ‘Zebra’ and 273 from A12359, were analyzed for segregation with 21 and 12 simple sequence repeat (SSR) markers, respectively. It is concluded that the inheritance mode of tetraploid bermudagrass was complete or near complete disomic. It is evident that the two bermudagrass parents had an allotetraploid genome with two distinct subgenomes since 33 SSR primer pairs amplified 34 loci, each having two alleles. Severe transmission ratio distortions occurred in the Zebra population while less so in the A12359 population. The findings of disomic inheritance and segregation ratio distortion in common bermudagrass is significant in subsequent linkage map construction, quantitative trait locus mapping and marker-assisted selection in the species.

## Introduction

Common bermudagrass [*C*. *dactylon* (L.) Pers. var. *dactylon*] is the best known and economically most important species in the genus *Cynodon* L. C. Rich. because of its widespread geographic distribution, important economic uses and enormous genetic variability [1,2]. The warm-season, sod-forming and perennial grass has been widely used for turf installation, forage production, soil stabilization and remediation in tropical and warmer temperate regions around the world [3]. In the United States, turf bermudagrass, including common bermudagrass and interspecific hybrids between common bermudagrass and African bermudagrass (*C*. *transvaalensis* Burtt-Davy), is a major warm-season turfgrass; whereas forage bermudagrass, encompassing common bermudagrass and interspecific hybrids between common bermudagrass and *C*. *nlemfuensis* Vanderyst, has been planted on approximately 12 million hectares as livestock herbage [4].

The knowledge of ploidy level and meiotic chromosome behavior is important for genetic research and breeding new cultivars [5]. Although the basic chromosome number of *Cynodon* species initially was mistakenly reported to be x = 10 [6] the confirmed basic chromosome number of *Cynodon* is x = 9 [7,8]. Among a series of ploidy levels (2n = 2x = 18, 3x = 27, 4x = 36, 5x = 45, and 6x = 54), tetraploid common bermudagrass is the most popular and prevalent form in nature [8–13]. During the meiosis of tetraploid common bermudagrass, chromosomes usually form 18 bivalents, but irregular pairing forms have been observed, including two or more univalents, or one or two quadrivalents [8, 14, 15]. Observing meioses of hybrids of 50 crosses within and between *C*. *dactylon* var. *dactylon* geographic races (tropical, temperate, and seleucidus), Harlan and de Wet indicated that most crosses that displayed bivalent pairing were found in hybrids between parents of similar geographic origins with exceptions [14].

The qualitative inheritance mode is essential information in a species not only because it illuminates homologous chromosome pairing behavior and transmission of alleles from parents to progenies, but also provides basic knowledge for linkage map construction, and marker-trait association like quantitative trait locus (QTL) mapping. The mode of allelic inheritance can also influence breeding procedures that are used for cultivar development. Bethel et al. reported that the tetraploid bermudagrass parent, T89, exhibited polysomic inheritance (with an autotetraploid genome) based on the ratio of 22 repulsion versus 102 coupling linkages revealed with single dose restriction fragment markers [16]. However, with the same mapping population, Harris-Shultz et al. indicated that T89 had two alleles segregating at each locus for 15 simple sequence repeat (SSR) loci except one progeny suggesting the parent displayed disomic inheritance and may be a segmental allotetraploid or allopolyploid rather than an autotetraploid [17]. Since tetraploid common bermudagrass is extremely diverse and widely distributed around the world [2,10], more work is needed to examine inheritance, segregation and genomic structure of the taxon. Therefore, our hypotheses were that the qualitative inheritance of tetraploid common bermudagrass was tetrasomic and that the species had an autopolyploid genome (i.e., two subgenomes were from the same diploid species). Accordingly, the specific objective of this study was to investigate the inheritance mode and segregation regularity in two common bermudagrass populations.

## Materials and Methods

### Plant materials

To facilitate codominant marker segregation analysis, two populations of first-generation selfed (S1) progenies were created and used to investigate the inheritance of common bermudagrass. The parent for one population was ‘Zebra’ (2n = 4x = 36) that was a variegated plant found in an F1 bermudagrass population growing on the Oklahoma State University Agronomy Research Farm at Stillwater, Oklahoma in 1971, and it was so named because its leaf blades had alternating green and chlorotic strips [18,19]. Zebra plants prepared in a greenhouse were moved into a growth chamber in the OSU Controlled Environment Research Laboratory (CERL) with 16 h/32.2°C light and 8 h/26.7°C dark periods in October and S1 seeds were harvested in December 2006. Seeds were germinated in another growth chamber in the winter between 2010 and 2011. A total of 228 single plants were transplanted into 10 cm pots in the greenhouse in the spring of 2011.

The second population consisted of selfed progenies of A12359 common bermudagrass (2n = 4x = 36) [10]. Originally, two parental plants, A12328 and A12359, were grown in alternating rows to make a full-sib hybrid population in an isolated plot on the OSU Agronomy Research Station. Mature inflorescences of A12359 were harvested from the plot in September, 2012 and February, 2013. Seeds were germinated in a growth chamber. Seedlings of A12359 were transplanted into 10 cm pots in the greenhouse. Since a small amount of seeds from A12328 were harvested, their seedlings were not included in subsequent genetic analysis. A total of 1275 progeny plants from A12359 seeds were developed and used for identification of selfed progeny with 12 polymorphic SSR primer pairs. A progeny was identified as a selfed progeny if all polymorphic bands of the 12 SSR primer pairs for the progeny were from A12359 and none from A12328. All progeny plants of the two S1 populations along with their respective parent plants were cultivated to maintain healthy growth in the greenhouse.

### DNA isolation and PCR conditions

Genomic DNA of each plant in the two populations and their parents was extracted following the cetyltrimethylammonium bromide (CTAB) method of Doyle with minor modifications [20]. Approximately 1.5 g of fresh leaf tissue from each plant was collected and ground to a fine powder in a Genogrinder (Zymo Research, CA, USA), and then transferred to a tube of pre-heated 1% CTAB buffer with 2-mercaptoethanol and incubated in 65°C water bath for 60 min. Chloroform/isoamyl alcohol was added for protein removal [5]. Iso-propanol was added to allow DNA to precipitate. The DNA pellet was washed with a washing buffer and stored in 1× TE Buffer (pH 8.0). The DNA concentration of each sample was quantified using a NanoDrop ND-1000 spectrophotometer (Nanodrop Products, DE, USA) and the DNA quality was checked by 0.5% KB^plus^ gels [21]. Each DNA sample was diluted to a concentration of 10 ng/μl for PCR (polymerase chain reaction).

Ninety-six-well PCR plates were used to perform SSR PCR reactions in a Biosystems 2720 Thermal Cycler (Applied Biosystems Inc., CA, USA). Each 10.5 μl reaction (one well) included 1.50 μl of 10 ng/ μl genomic DNA, 4.87 μl nuclease free water, 1.00 μl 1× standard *Taq* reaction buffer (10 mM Tris-HCl, 50 mM KCl and 1.5 mM MgCl_2_) (New England Biolabs Inc., MA, USA), 0.20 μl 10 mM dNTPs (Promega Corporation, WI, USA), 0.05 μl *Taq* enzyme (0.025 Units) (New England Biolabs Inc., MA, USA), 0.20 μl 1 μM IR-700 or -800 fluorescence labeled M 13 primer, and 1.34 μl each of 10 μM/μl forward and reverse primers. The PCR cycling was started with a denaturing step at 95°C for 5 min; followed by 14 cycles of 94°C for 20 s, 58°C 1 min, and 72°C 30 s; 28 cycles of 94°C for 20 s, 55°C 1 min, and 72°C 30 s; and a final extension at 72°C for 10 min, and then was stored at 4°C. A Blue Stop Solution (95% formamide, 25mM ethylenediaminetetraacetic acid, and 2% bromophenol blue) of 5.0 μl was added to each PCR reaction bringing a total volume to 15.5 μl, which was denatured for 3 min at 94°C [5]. The PCR products in two plates were labeled with 700 nm and 800 nm fluorescence primers respectively, were mixed thoroughly, spun down, and loaded into wells of 6.5% KB^plus^ gels for electrophoresis on a 4300 LI-COR DNA Analyzer (LI-COR Biosciences, Lincoln, NE). Parameters for the electrophoresis in the LI-COR 4300 DNA Analyzer were set at 1500 V, 40 mA, 40 W, 45°C and 15 min for a pre-run, and one hour and 45 min to finish rest of the run.

### Polymorphic SSR primer pair selection, gel scoring and data analysis

For each of the two populations, a small subset, which included two replicates of the parent and six randomly selected progeny samples, was used to screen common bermudagrass SSR primer pairs developed in our lab in order to select 33 polymorphic pairs (S1 Table). If two or more bands were amplified with an SSR primer pair and heritable in the subset, the SSR primer pair was judged to be polymorphic [21]. For a parent with “Aa” bands, if only one upper band was amplified in a progeny sample, the band pattern was scored as “AA”; if only one lower band amplified it was scored as “aa”; and if two bands amplified it was scored as “Aa”. If four bands were amplified for a parent, the band pattern was scored as “abcd”, and if three bands of a progeny were amplified, it was scored as “abc”, or “abd”, or “acd”, or “bcd” depending on the size of each band (“a” > “b” > “c” > “d”). If a PCR failed or an ambiguous band was found for a sample, it was scored as “M” (i.e., missing) [5].

Chi-square test was used to examine inheritance modes of SSR markers in the two populations. Segregation ratios of SSR markers in each of the two populations were tested to determine segregation modes, disomic versus tetrasomic inheritance. For a polymorphic SSR locus under disomic inheritance, if a parental genotype was *Aa* which would have an “Aa” band pattern, then the progeny segregation ratio would be 1AA: 2Aa: 1aa (Table 1). Under tetrasomic inheritance, if the parent had “Aa” bands, there were three possible genotypes, *AAAa*, or *AAaa*, or *Aaaa*, the expected phenotype segregation ratio in the progenies would be 1 upper band (AAAA): 3 double bands (2AAAa + 1AAaa), or 1 upper band (AAAA): 34 double bands (8AAAa + 16AAaa + 2AAaa + 8Aaaa): 1 lower band (aaaa), or 3 double bands (1AAaa + 2Aaaa):1 lower band (aaaa), respectively. If one SSR primer pair amplified four bands, under tetrasomic inheritance, the parent would have a genotype *ABCD*, and the expected progeny band patterns and segregation ratio are given in Table 2. The software “Calculation for the chi-square test: An interactive calculation tool for chi-square tests of goodness of fit and independence” was used to perform chi-square tests and calculate actual P-values [22].

**Table 1:** Twenty-one SSR markers and their segregation data in 228 selfed progenies of ‘Zebra’ common bermudagrass.

SSR marker information		Chi-square testing in 228 selfed progenies							
		Observed progeny bands				For 1:2:1 ratio (disomic)		For 1:34:1 ratio (tetrasomic)	
No.	Primer pair ID	AA	Aa	aa	Missing	χ^2^	*P*	χ ^2^	*P*
1	CDCA1-21/22	16	183	28	1	86.38	<0.01	94.28	<0.01
2	CDCA2-231/232	39	111	77	1	12.38	<0.01	1013.01	<0.01
3	CDCA3-247/248	50	147	28	3	25.46	<0.01	402.13	<0.01
4	CDCA3-313/314	10	132	86	0	56.35	<0.01	1042.73	<0.01
5	CDCA4-323/324	16	156	47	9	48.27	<0.01	309.40	<0.01
6	CDCA7-653/654	39	110	77	2	12.94	<0.01	1013.24	<0.01
7	CDCA7-693/694	13	120	93	2	57.50	<0.01	1241.16	<0.01
8	CDCA8-737/738	31	146	47	4	22.93	<0.01	388.03	<0.01
9	CDGA1-847/848	37	135	56	0	10.90	<0.01	571.69	<0.01
10	CDGA1-921/922	52	100	73	3	6.70	0.04	1107.34	<0.01
11	CDGA1-929/930	8	161	52	7	63.68	<0.01	356.91	<0.01
12	CDGA3-1103/1104	10	173	42	3	74.17	<0.01	214.08	<0.01
13	CDGA4-1245/1246	36	184	7	1	94.99	<0.01	144.40	<0.01
14	CDGA5-1427/1428	60	153	15	0	44.45	<0.01	487.82	<0.01
15	CDGA7-1611/1612	0	172	56	0	86.53	<0.01	407.12	<0.01
16	CDGA8-1765/1766	48	117	61	2	1.78	0.41	794.50	<0.01
17	CDGA8-1807/1808	44	117	61	6	3.25	0.19	755.73	<0.01
18	CDATG6-2123/2124	9	145	71	3	52.95	<0.01	693.46	<0.01
19	CDATG6-2143/2144	57	154	15	2	45.36	<0.01	436.56	<0.01
20	CDAAC7-2703/2704	37	161	28	2	41.50	<0.01	237.21	<0.01
21	CDCAG3-2897/2898	1	174	51	2	87.98	<0.01	328.89	<0.01

**Table 2:** Counts of observed and expected phenotypes amplified with SSR CDAAC7-2693/2694 in 272 S1 progenies of A12359 common bermudagrass under tetrasomic and disomic inheritance.

Observed		Expected genotypes and phenotypes of 272 progenies if			
		Parent genotype *ABCD* at one locus under tetrasomic inheritance		Parent genotype of two independent loci *AB* & *CD* under disomic inheritance§	
Phenotypes	Counts	Genotypes	Counts	Genotypes	Counts
ab	0	*AABB*	7.5	*AABB*	0
abc	3	*AABC*, *ABBC*, *ABCC*	45.4	*ABCC*	34
abd	45	*AABD*, *ABDD*, *ABDD*	45.4	*ABDD*	34
abcd	77	*ABCD*	45.4	*ABCD*	68
ac	9	*AACC*	7.5	*AACC*	17
acd	69	*AACD*, *ACCD*, *ACDD*	45.4	*AACD*	34
ad	26	*AADD*	7.5	*AADD*	17
bc	2	*BBCC*	7.5	*BBCC*	17
bcd	21	*BBCD*, *BCCD*, *BCDD*	45.4	*BBCD*	34
bd	20	*BBDD*	7.5	*BBDD*	17
cd	0	*CCDD*	7.5	*CCDD*	0

^§^: AB and CD were considered respective genotypes at two separate loci based on the appearance of “ac”, “ad”, “bc” and “bd” phenotypes in the progeny population.

## Results

### Selection of polymorphic SSR markers and verification of selfed progeny

Fifty-one common bermudagrass SSR PPs (details not provided) were initially screened for polymorphism in the subset of Zebra population, resulting in selecting of 21 SSRs for the further genotyping work (Table 1). This was because each of the 21 selected SSR primer pairs produced polymorphic, heritable bands, while the other 30 amplified single bands or unclear bands, thus, were discarded. Similarly, 12 polymorphic SSRs were selected to genotype the A12359 S1 population (S1, S2 and S3 Tables, and Table 3). All 228 progeny plants derived from Zebra were selfed progenies while 273 progeny plants derived from A12359 were selfed as identified using the respective polymorphic SSR markers. Hence, the selfed plants formed two separate S1 populations, one from Zebra and the other from A12359 used in subsequent analyses.

**Table 3:** Twelve SSR markers and their segregation in 273 selfed progenies of parent A12359 common bermudagrass.

SSR marker		Chi-square testing in 273 selfed progenies							
		Progeny genotypes				For 1:2:1 (disomic)		For 1:34:1 (tetrasomic)	
No.	Primer pair ID	AA	Aa	aa	Missing	χ^2^	P	χ ^2^	P
1	CDCA5-491/492	67	126	73	7	1.01	0.6	1106.32	<0.01
2	CDCA7-623/624	60	154	54	5	6.24	0.04	694.54	<0.01
3	CDGA1-783/784	57	152	62	2	4.2	0.12	764.98	<0.01
4	CDGA3-1195/1196	62	125	70	16	0.69	0.71	1021.85	<0.01
5	CDGA6-1583/1584	47	152	65	9	8.51	0.01	686.65	<0.01
6	CDGA7-1601/1602	4	195	66	8	87.97	<0.01	470.03	<0.01
7	CDGA8-1795/1796	70	143	58	2	1.89	0.39	910.75	<0.01
8	CDATG1-1889/1890	66	117	83	7	6.02	0.05	1287.87	<0.01
9	CDATG3-1999/2000	59	145	59	10	2.77	0.25	750.05	<0.01
10	CDAAC5-2523/2524	66	139	67	1	0.14	0.93	982.51	<0.01
11	CDAAC7-2675/2676	67	141	59	6	1.32	0.52	874.56	<0.01
12	CDAAC7-2693/2694 (1)	103	117	43	10	30.57	<0.01	1453.26	<0.01
	CDAAC7-2693/2694 (2)	14	168	90	1	57.53	<0.01	943.95	<0.01

### Genotyping of selected SSR markers

**Fig 1:**
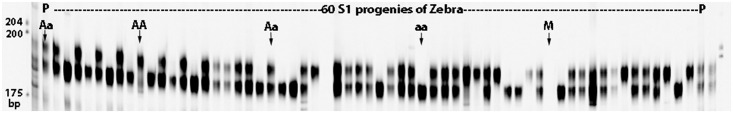
A gel image of ‘Zebra’ common bermudagrass and its 60 progenies amplified with SSR CDGA8-1765/1766. The band pattern of Zebra (P) was coded as “Aa”, and a progeny band pattern coded as “AA” if one upper band, “Aa” if two bands, and “aa” if one lower band. A missing lane was labeled as ‘M’. Standard size markers were loaded on first and last lanes.

**Fig 2:**
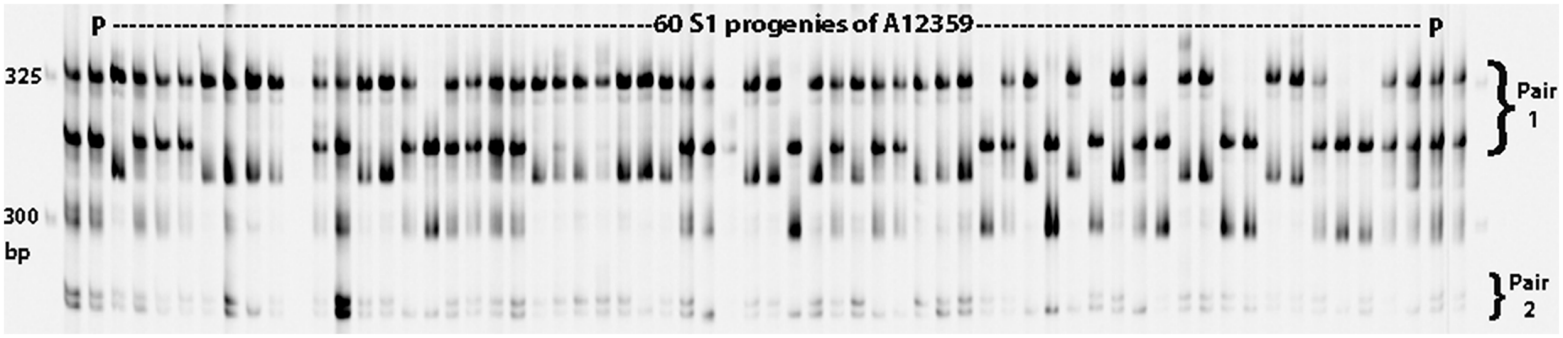
A gel image of A12359 and its 60 progenies amplified with an SSR CDAAC7-2693/2694. Four bands of A12359 segregated in two pairs labeled on the right side of the gel, “Pair 1” for bands in the range of 300 and 325 bp, and “Pair 2” for the other pair smaller than 300 bp. P represents the parent A12359 common bermudagrass. Size markers are labeled for the first lane on the left side.

The Zebra S1 progenies were genotyped with 21 selected polymorphic SSRs (Table 1), and all of them consistently amplified two segregating bands (Fig 1 and Table 1). This result suggests the genotypes of Zebra are likely *Aa* following disomic inheritance, which was based on the assumption if each SSR only amplified one locus in one subgenome, rather than in both subgenomes. Another assumption for an SSR with two segregating bands was that the genotype of Zebra was either of *Aaaa*, *AAaa* or *AAAa* under tetrasomic inheritance, which was derived from amplifying one locus by one SSR. Three or four segregating bands per SSR locus were not observed although they were expected in some SSR loci in tetrasomic inheritance. Among the 12 polymorphic SSR markers genotyped in the A12359 S1 population, 11 produced two segregating bands while one (CDAAC7-2693/2694) amplified four segregating bands (Fig 2 and Table 3). Under disomic inheritance, the four bands of A12359 would represent two independent loci (i.e., *AB* genotype at one locus, and *CD* at the other), while the genotype would be *ABCD* at one locus under tetrasomic inheritance (S4 and S5 Tables).

### Segregation analysis

In the Zebra S1 population, chi-square tests indicated that the segregation of two SSRs (No. 16 and 17) was consistent with the 1:2:1 Mendelian segregation ratio (P = 0.41 and 0.19)for disomic inheritance (Table 1). One SSR (No. 15) produced a segregation pattern following a 3Aa: 1aa (P = 0.88)that was expected for the segregation of an *Aaaa* genotype under tetrasomic inheritance. The segregation patterns of the 18 other SSRs were much closer to the 1:2:1 for disomic inheritance ratio than to 1:34:1 for tetrasomic inheritance based on P values in chi-square testing, although their patterns did not fit either of them at p<0.001 (Table 1).

For the A12359 S1 population, all the 12 polymorphic SSRs except CDAAC7-2693/2694 amplified two bands in the parent, and segregated in its selfed progenies (Table 3). Segregation of eight SSRs in the population followed the 1:2:1 disomic segregation ratio (P≥0.05) and two SSRs had a segregation ratio close to 1:2:1 (0.05>P≥0.01), while the segregation of CDGA7-1601/1602 was significantly deviated from the disomic ratio (P<0.01). It is worth noting that none of these 11 SSR markers exhibited segregation approaching the 1:34:1 theoretical ratio (Table 3)for selfing a tetrasomic *AAaa* genotype.

## Discussion

Under disomic inheritance, there would be possibilities that one SSR amplified one locus on subgenome *A* and another locus on subgenome *B*, resulting in two bands for the parent if the genotypes of the parent were *AA/Bb*, *AA/bb*, *Aa/BB*, *Aa*/*Bb*, *Aa*/*bb*, *aa*/*BB*, or *aa*/*Bb* [23] and if both A and B alleles have the same band size (i.e., A = B) and both a and b alleles have the same band size (a = b) (S6 Table). The expected segregation ratios in S1 progenies would be 1 upper band: 3 double bands for *AA/Bb* and *Aa/BB*; all double bands (i.e., no segregation) for *AA/bb* and *aa*/*BB*; 1 upper band: 14 double bands: 1 lower band for *Aa*/*Bb*; 3 double bands: 1 lower band for *Aa*/*bb* and *aa*/*Bb*, respectively. The observed segregation patterns in the two populations (Tables 1 and 3) were not consistent with the expected ratios of tetrasomic inheritance except 3:1. In addition, the theoretical frequency of the 3:1 ratio would be 4 of 7 (58%), but we only observed 1 out of 33 (3%) SSRs in the two populations. Therefore, there was no possibility that one locus was amplified in each subgenome, and band size of each allele was the same for any observed SSRs in our common bermudagrass populations.

Interestingly, the SSR CDAAC7-2693/2694 amplified four bands, scored as “abcd” in A12359 and segregated in the S1 population (Fig 2 and Table 2). Under tetrasomic inheritance, a parent with the “abcd” band pattern would have *ABCD* genotype and produce 19 genotypes resulting in 11 phenotypes in its progenies (Table 2). In contrast, under disomic inheritance, two independent loci of *AB* at one locus and *CD* at the other would produce an S1 population of nine phenotypes (Table 2). Obviously, nine phenotypes observed in A12359 S1 population were perfectly consistent with that of expected phenotypes if the two loci segregated independently in disomic inheritance, and other two phenotypes with band patterns “ab” and “cd” which were expected under tetrasomic inheritance were not observed in the S1 progenies for CDAAC7-2693/2694. Using the pooled data in Table 2, Chi-square testing indicated that the segregation of alleles of the SSR in A12359 S1 population significantly differed from tetrasomic inheritance (χ^2^ = 172.8, P<0.0001) and also deviated from disomic inheritance (χ^2^ = 96.3, P<0.0001). Further chi-square testing for segregation data at each of the two loci revealed that neither followed the typical 1:2:1 ratio (Table 3).

The inheritance mode of two tetraploid common bermudagrasses was analyzed with 33 SSRs amplifying 34 loci in two S1 populations. Transmission of two target bands at each locus and segregation ratios are consistent with preferential pairing at 33 loci exhibiting disomic inheritance. The segregation pattern of two alleles at the locus amplified by SSR CDGA3-1611/1612 was not different from a 3Aa:1aa ratio, suggesting either disomic or tetrasomic inheritance. For tetrasomic inheritance, the cytological observations in the past indicated that four chromosomes pairing formed one or two quadrivalents in some genotypes of common bermudagrass [8,14,15]. However, the 3Aa: 1aa segregation could be a result of segregation ratio distortion if all homozygous “*AA*” plants were dead.

Of the 33 SSRs tested in the two S1 populations, only one amplified four alleles, which segregated at two separate loci. The results indicated substantial subgenome differentiation in common bermudagrass, revealing common bermudagrass is an allotetraploid. However, the finding was not expected as it is opposite to the previously proposed hypothesis that common bermudagrass was an autotetraploid [14,24]. Harlan and de Wet [14] indicated that diploid *C*. *dactylon* var. *aridus* Harlan et de Wet (2n = 2x = 18) bermudagrass was the only likely source of tetraploid common bermudagrass. Var. *aridus* is the only diploid bermudagrass which has rhizomes and is morphologically similar to tetraploid common bermudagrass. It is warranted to confirm this finding by genotyping a larger number of codominant markers (i.e., SSRs) that cover the whole genome in the cosmopolitan common bermudagrass.

Segregation distortions were evident in the two S1 populations. Nineteen of 21 (90%) loci in the Zebra S1 population and four of 13 (31%) loci in the A12359 population had distorted segregation ratios. Segregation distortion is common in allogamous species, especially when they are selfed. Liu et al. reported segregation ratio distortion was identified for approximately 19% of loci in an S1 population of switchgrass (*Panicum virgatum* L.) [25], while Okada et al. observed segregation ratio distortion for 14% of loci in a hybrid population in the species [26]. Selfed populations tend to have a higher segregation ratio distortion because of inbreeding depression [27,28]. Segregation ratio distortion occurs when a molecular marker locus links to a distorter, such as recessive lethal genes [29]. When the recessive lethal genes become homozygous under selfing or inbreeding, the plant is weak or dead.

Segregation distortion can also cause spurious linkages and biased recombination fractions leading to inaccurate genetic distances between markers and incorrect marker order of linkage maps [25]. Subsequently, quantitative trait locus mapping is negatively affected if an inaccurate linkage map is used. One strategy commonly employed in construction of linkage maps is to use regularly segregated markers to establish a frame map before attaching segregation distorted markers [25,26,30]. Another strategy is to use a population which has fewer segregation distortions for linkage mapping (in this case using A12359 S1 population rather than the Zebra S1 population).

## Conclusions

In summary, we report two common bermudagrass populations exhibiting complete or near complete disomic inheritance and provide evidence supporting two distinct subgenomes constituting an allotetraploid genome in two tetraploid genotypes (Zebra and A12359). Severe to moderate segregation distortion occurred in the two S1 populations. The findings add to the knowledge pool of genetics and will benefit genetic map construction, quantitative trait locus mapping and breeding efforts in the taxon, *C*. *dactylon* var. *dactylon*.

## Supporting Information

S1 Table Information for selected SSR primer pairs in genotyping two selfed populations in common bermudagrass. (DOCX)

S2 Table Genotype data for Zebra selfed population. (XLS)

S3 Table Genotype data for A12359 selfed population (XLSX)

S4 Table Possible genotypes of gametes and zygotes under tetrasomic inheritance if the parental genotype is *ABCD* at one locus. (DOCX)

S5 Table Possible genotypes of gametes and zygotes under disomic inheritance if the parental genotype is *AB* & *CD* at two independent loci. (DOCX)

S6 Table Genotypes of gametes and zygotes for possible parental genotypes under disomic inheritance if one SSR primer pair simultaneously amplifies one locus in both subgenomes. (DOCX)
