# Supplementary material for: Disomic Inheritance and Segregation Distortion of SSR Markers in Two Populations of Cynodon dactylon (L.) Pers. var. dactylon
Source: PLoS One. 2015 Aug 21;10(8):e0136332. doi: 10.1371/journal.pone.0136332 (PMC4546580; doi:10.1371/journal.pone.0136332)
Supplement: S1 Table — (DOCX) [file pone.0136332.s001.docx]

**Supplemental Table 1. Information for selected SSR primer pairs in genotyping two selfed populations in common bermudagrass.**

| No. | Marker ID | Repeat Motif | Primer Sequence  (5’ to 3’) | Expected Size Plus M13F (bases) |
| --- | --- | --- | --- | --- |
| 1 | CDCA1-21/22 | (AC)13-(AG)14 | F: GGGCCTCCCCTTTTATACAT  R: GGTAACCAATCAAGGCCACT | 199 |
| 2 | CDCA2-231/232 | (CA)13 | F: CTTTTACGTCGGCCTTTGAG  R: GTCCTGGTCTTGTCGGTTTC | 186 |
| 3 | CDCA3-247/248 | (GT)8 | F: TACCTCGCTGGACTGAAGTG  R: GCAGTAGTCCCACCAACCTT | 190 |
| 4 | CDCA3-313/314 | (CA)23 | F: GGGTCATGAGTCAAATGTGC  R: CTTTTGTGAGCCAGAAGCAA | 311 |
| 5 | CDCA4-323/324 | (TG)13 | F: ACAAGCCTTCCGGACTTTTA  R: CATGCAGCACTGCTCAGATA | 237 |
| 6 | CDCA5-491/492 | (GT)12 | F: CTTGGTTCTTGGGTCCTTGT  R: AGCTCAAGCACCATTGTCAG | 289 |
| 7 | CDCA7-623/624 | (AC)17-(CA)5 | F: CGAGACCTAGTGAACAGCGA  R: GGCCGTGCTTAAAGGAATAG | 329 |
| 8 | CDCA7-653/654 | (TG)10-(AG)9 | F: CGCTACACTTTCGAGGTCAA  R: ACCAGATATGCACTCCACCA | 165 |
| 9 | CDCA7-693/694 | (AC)11 | F: CGTCATCACAACCCACTCTC  R: TGTTCTTCCACGACTGCTTC | 160 |
| 10 | CDCA8-737/738 | (TG)14-(AG)11 | F: ATGTCTAAAACACGCCCACA  R: GACAACCAAGAGTGGCGATA | 338 |
| 11 | CDGA1-783/784 | (GA)14 | F: CACTGTTTACCCATCCAACG  R: TTTTCGTACACACCCCAGAA | 240 |
| 12 | CDGA1-847/848 | (CT)19-(TC)5-(TG)8 | F: CCGATCGCTACTGAGAAACA  R: TGGCCGAAAAACAGGGTA | 296 |
| 13 | CDGA1-921/922 | (GA)14 | F: GTTGGGTGAACGTACACAGG  R: TAATTGACGTCCCTTCCCTC | 267 |
| 14 | CDGA1-929/930 | (GA)18 | F: TCAAGGTACCTGATGTGGAAAC  R: GACTTCCCCTTAACAGCAGC | 162 |
| 15 | CDGA3-1103/1104 | (AG)16 | F: AAGAATAATGCCCAAGGCAC  R: ACCATCACTCGACACCACAT | 258 |
| 16 | CDGA3-1195/1196 | (CT)15 | F: ACCACCAATAGCACACCAGA  R: CGGAACAAGGAGTGAGACAC | 324 |
| 17 | CDGA4-1245/1246 | (GA)20 | F: AAGGAAAGGTGCATACCTGG  R: GGCAGGTGTGGAGAAGTACA | 194 |
| 18 | CDGA5-1427/1428 | (CT)21 | F: TAGCAGGAACCTGTGGTCTG  R: TGTTCTAACTGTCGCCATCC | 307 |
| 19 | CDGA6-1583/1584 | (AG)25 | F: GTATCGTCATCGTCCTGGTG  R: TCGGCCAGAAAACCTCTATT | 349 |
| 20 | CDGA7-1601/1602 | (GA)13 | F: CCTGCTGGTCAGAACTCAAC  R: TATTGGTTGCACCTTCCAGA | 257 |
| 21 | CDGA7-1611/1612 | (AGAT)6-(AG)15 | F: TCCTTCTTGTCCTGAAGCCT  R: ACAGTCCATGCGACTCAGAA | 246 |
| 22 | CDGA8-1765/1766 | (TC)16 | F: GGGCTTTTGGAATGACTTGT  R: CGAAGAGCGAGGAGAGATTT | 198 |
| 23 | CDGA8-1795/1796 | (AC)5-(AG)36 | F: TTCGTGGACTCTGGCTATTG  R: GCCCAGGTAACGTGTTCTTT | 364 |
| 24 | CDGA8-1807/1808 | (GA)14 | F: CCTCAACTCCAGTGCTGAAA  R: TGTTAACCGGGGTTCAGATT | 226 |
| 25 | CDATG1-1889/1890 | (GCT)7-(GAT)7 | F: AAACGTGAGAGGCTCTTGCT  R: GTATGACACACGGAAGGACG | 309 |
| 26 | CDATG3-1999/2000 | (ATG)7 | F: CCAGGTTCGCATCAGATA  R: TGCATATCATGAACACGACG | 278 |
| 27 | CDATG6-2123/2124 | (CAT)5 | F: AATGGAACCTTGGCACTTTC  R: GGTGGGTGTTACTGCTCCTT | 197 |
| 28 | CDATG6-2143/2144 | (TCT)6 | F: ATCCTTCCCCTCCTCTTTGT  R: TTGTACGATATCAACCCGGA | 355 |
| 29 | CDAAC5-2523/2524 | (TGT)9 | F: AAGGCCTAACCCAATTTGC  R: ACAATGCTTTTCATCCTCCC | 214 |
| 30 | CDAAC7-2675/2676 | (TGT)8 | F: TAGCCTACCCCAACTTGCTT  R: GTATACTGGCTTCATGGGCA | 206 |
| 31 | CDAAC7-2693/2694 | (AAC)7 | F: TTGCCTACCAAACACGAAAG  R: TCCAAACTCGTGTAATTGCC | 321 |
| 32 | CDAAC7-2703/2704 | (ACA)10 | F: CTATTGCACATTGGATTCCG  R: AGGAGTGGGAGGGTTTCTTT | 359 |
| 33 | CDCAG3-2897/2898 | (CTG)7 | F: TTGCCACTTTTGCAGGTAAC  R: AAGTAGTGCCATGCGATCAG | 174 |

Marker ID, repeat motif, primer sequences and expected band size for each of 33 selected SSR primer pairs used for genotyping Zebra and A12359 first-generation selfed populations.
