# Supplementary material for: Disomic Inheritance and Segregation Distortion of SSR Markers in Two Populations of Cynodon dactylon (L.) Pers. var. dactylon
Source: PLoS One. 2015 Aug 21;10(8):e0136332. doi: 10.1371/journal.pone.0136332 (PMC4546580; doi:10.1371/journal.pone.0136332)
Supplement: S4 Table — (DOCX) [file pone.0136332.s004.docx]

**S4 Table. Possible genotypes of gametes and zygotes under tetrasomic inheritance if the parental genotype is *ABCD* at one locus.**

|  | *AB* | *AC* | *AD* | *BC* | *BD* | *CD* |
| --- | --- | --- | --- | --- | --- | --- |
| *AB* | *AABB* | *AABC* | *AABD* | *ABBC* | *ABBD* | *ABCD* |
| *AC* | *AABC* | *AACC* | *AACD* | *ABCC* | *ABCD* | *ACCD* |
| *AD* | *AABD* | *AACD* | *AADD* | *ABCD* | *ABDD* | *ACDD* |
| *BC* | *ABBC* | *ABCC* | *ABCD* | *BBCC* | *BBCD* | *BCCD* |
| *BD* | *ABBD* | *ABCD* | *ABDD* | *BBCD* | *BBDD* | *BCDD* |
| *CD* | *ABCD* | *ACCD* | *ACDD* | *BCCD* | *BCDD* | *CCDD* |
