# Supplementary material for: Disomic Inheritance and Segregation Distortion of SSR Markers in Two Populations of Cynodon dactylon (L.) Pers. var. dactylon
Source: PLoS One. 2015 Aug 21;10(8):e0136332. doi: 10.1371/journal.pone.0136332 (PMC4546580; doi:10.1371/journal.pone.0136332)
Supplement: S5 Table — (DOCX) [file pone.0136332.s005.docx]

**S5 Table. Possible genotypes of gametes and zygotes under disomic inheritance if the parental genotype is *AB* & *CD* at two independent loci**

|  | *AC* | *AD* | *BC* | *BD* |
| --- | --- | --- | --- | --- |
| *AC* | *AACC* | *AACD* | *ABCC* | *ABCD* |
| *AD* | *AACD* | *AADD* | *ABCD* | *ABDD* |
| *BC* | *ABCC* | *ABCD* | *BBCC* | *BBCD* |
| *BD* | *ABCD* | *ABDD* | *BBCD* | *BBDD* |
