# Supplementary material for: Disomic Inheritance and Segregation Distortion of SSR Markers in Two Populations of Cynodon dactylon (L.) Pers. var. dactylon
Source: PLoS One. 2015 Aug 21;10(8):e0136332. doi: 10.1371/journal.pone.0136332 (PMC4546580; doi:10.1371/journal.pone.0136332)
Supplement: S6 Table — (DOCX) [file pone.0136332.s006.docx]

**S6 Table. Genotypes of gametes and zygotes for possible parental genotypes under disomic inheritance if one SSR primer pair simultaneously amplifies one locus in both subgenomes**

Parental genotype: *AA/Bb*

|  | *AB* | *Ab* |
| --- | --- | --- |
| *AB* | *AABB* | *AABb* |
| *Ab* | *AABb* | *AAbb* |

Parental genotype: *AA/bb*

|  | *Ab* |
| --- | --- |
| *Ab* | *AAbb* |

Parental genotype: *Aa/BB*

|  | *AB* | *aB* |
| --- | --- | --- |
| *AB* | *AABB* | *AaBB* |
| *aB* | *AaBB* | *aaBB* |

Parental genotype: *Aa/Bb*

|  | *AB* | *Ab* | *aB* | *ab* |
| --- | --- | --- | --- | --- |
| *AB* | *AABB* | *AABb* | *AaBB* | *AaBb* |
| *Ab* | *AABb* | *AAbb* | *AaBb* | *Aabb* |
| *aB* | *AaBB* | *AaBb* | *aaBB* | *aaBb* |
| *ab* | *AaBb* | *Aabb* | *aaBb* | *aabb* |

Parental genotype: *Aa/bb*

|  | *Ab* | *ab* |
| --- | --- | --- |
| *Ab* | *AAbb* | *Aabb* |
| *ab* | *Aabb* | *aabb* |

Parental genotype: *aa/BB*

|  | *aB* |
| --- | --- |
| *aB* | *aaBB* |

Parental genotype: aa/Bb

|  | *aB* | *ab* |
| --- | --- | --- |
| *aB* | *aaBB* | *aaBb* |
| *ab* | *aaBb* | *aabb* |
